# Characterization of the transcriptomes and cuticular protein gene expression of alate adult, brachypterous neotenic and adultoid reproductives of *Reticulitermes labralis*

**DOI:** 10.1038/srep34183

**Published:** 2016-09-30

**Authors:** Xiaohong Su, He Liu, Xiaojuan Yang, Jiaoling Chen, Honggui Zhang, Lianxi Xing, Xiaojing Zhang

**Affiliations:** 1 Key Laboratory of Resource Biology and Biotechnology in Western China, Ministry of Education, Northwest University, Xi’an, China.; 2 Shaanxi Key Laboratory for Animal Conservation, Northwest University, Xi’an, China; 3 College of Life Sciences, Northwest University, Xi’an, China

## Abstract

The separation of primary reproductive and secondary reproductive roles based on the differentiation of alate adults and neotenic reproductives is the most prominent characteristic of termites. To clarify the mechanism underlying this differentiation, we sequenced the transcriptomes of alate adults (ARs), brachypterous neotenics (BNs) and adultoid reproductives (ANs) from the last instar nymphs of *Reticulitermes labralis*. A total of 404,152,188 clean sequencing reads was obtained and 61,953 unigenes were assembled. Of the 54 identified cuticular protein (CP) genes of the reproductives, 22 were classified into the CPR family and 7 were classified into the CPG family. qRT-PCR analyses of the 6 CP genes revealed that the CP genes involved in exocuticle sclerotization were highly expressed in the ARs and RR-1 involved in soft endocuticle was highly expressed in the ARs and ANs. These results suggest that the alate adults might increase cuticular component deposition to adapt to new or changing environments and that the development of reproductive individuals into primary or secondary reproductives is controlled by the expression of cuticular protein genes involved in the hardening of the exocuticle. In addition, the AN caste is a transitional type between the BN and AR castes in the process of evolution.

Caste differentiation in termites is one of the most conspicuous examples of facultative polyphenism in animals. Facultative polyphenism is the phenomenon in which individuals exhibit various phenotypes despite sharing the same genetic background^1^. The reproductive caste of many termite species, especially *Reticulitermes*, exhibits remarkable plasticity. Primary reproductives develop via the moulting of the last instar nymphs into winged sexuals (alate adults) that shed their wings after the nuptial flight and create new colonies in the absence of workers. In the absence of or at a great distance from the primary reproductives, a few of the nymphs or workers develop into secondary reproductives to provide for the continued or additional growth of the colony^1^,^2^. In *R. urbis* and *R. labralis,* the last instar nymphs can develop in three manners: (i) via a single moult into alate adults as primary reproductives, (ii) via a single moult into brachypterous neotenics as secondary reproductives, and (iii) via a single moult into adultoid reproductives with floppy wings as secondary reproductives^3^,^4^,^5^. Each reproductive caste exhibits a particular behavioural repertoire and caste-specific morphological characteristics depending on the environmental and social stimuli^2^. This reproductive system of termites is considered to be the basis for the evolutionary and ecological success of these eusocial insects.

The differentiation of reproductive castes in termites is a multifaceted process that is under the control of a range of intrinsic and extrinsic factors. Advances in molecular, genomic, and integrative or “systems” biology in the past decade have greatly facilitated efforts to begin to understand this process. Termite scientists have confirmed that differential gene expression occurs between reproductives, workers and soldiers as well as during the course of caste differentiation. In recent years, next-generation high-throughput DNA sequencing techniques have provided fascinating opportunities in the life sciences and have dramatically improved the efficiency and cost effectiveness of gene discovery. Next-generation sequencing generates large amounts of data and, for this reason, has been applied to the study of termites. Huang *et al*.^6^ sequenced the transcriptome of *Odontotermes formosanus* (Shiraki) workers and identified the expression levels of three genes involved in the differentiation of workers and soldiers^6^. Husseneder *et al*.^7^ profiled the transcriptomes of female alates and egg-laying queens of the Formosan subterranean termite *Coptotermes formosanus* (Shiraki) and described the changes in transcription that occur when virgin alates become egg-laying queens^7^. However, the changes in the transcriptome that are involved in the differentiation of the primary and secondary reproductive castes of termite have not been studied.

The cuticle of secondary reproductives is less sclerotized than that of primary reproductives, which is the most significant morphological difference between these reproductives^1^. The insect exoskeleton is a remarkable extracellular structure that is secreted by epidermal cells and serves as the outer body covering. The exoskeleton helps to protect the insect from environmental stresses, such as predators, parasites, abrasions, desiccation, and UV radiation. The exoskeleton functions as an attachment site for the internal muscles and organs and is instrumental to locomotion and flight^8^,^9^. Also, the exoskeleton have a role in chemical communication and therefore sociality by hosting cuticular hydrocarbons. Structural cuticular proteins (CPs) and polysaccharide chitin are the major components of the exo- and endocuticular layers. The expression of specific CPs is probably required for the formation of diverse cuticles at different developmental stages that exhibit appropriate combinations of physical and morphological properties to provide structural support, mechanical protection and mobility^10^. Termite cuticular armament is thin and less sclerotized than that of other social insects, which renders termites more vulnerable to parasites that invade through the integument. Interestingly, although the last instar nymphs of *R. labralis* develop into alate adults, brachypterous neotenics and adultoid reproductives through a single moult, only the alate adults have well-sclerotized and pigmented cuticles, and a pair of alate adults can found a new colony without the help of workers. Therefore, we speculate that the CP characteristics might determine whether a reproductive individual can or cannot become a primary reproductive.

The separation of the primary reproductive and secondary reproductive roles based on alate adult and neotenic reproductive differentiation is the most prominent characteristic of termites. To clarify the mechanism underlying this differentiation in the present study, we sequenced the transcriptome of the three different reproductive castes, that is, alate adults (ARs), adultoid reproductives (ANs) and brachypterous neotenics (BNs), from the last instar nymphs of *R. labralis* and subsequently quantified the differential expressions of six cuticular protein genes via quantitative real-time reverse transcription PCR (qRT-PCR). The expression profiles of the cuticular protein genes in primary and secondary reproductives will be addressed in the present study.

## Results and Discussion

### Differences in the morphologies of the ARs, ANs and BNs

**Figure 1:**
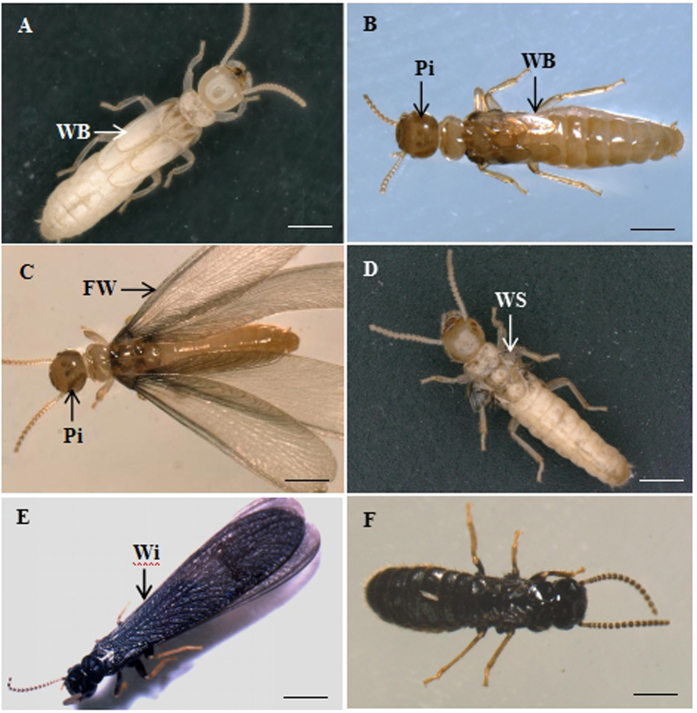
The morphologies of the BNs, ANs and ARs. (**A**) The last instar nymphs had a white body and long wing buds. (**B**) The BNs had wing buds, a light brown cuticle and dark brown stripes on their heads. (**C**) The ANs had floppy wings and a light brown body. (**D**) The floppy wings of ANs were shed and left two pairs of wing scales. (**E**) The ARs were characterized by darkened pigmentation, a hard cuticle and black wings. (**F**) The wings of ARs were shed. WB, wing buds; Pi, pigmentation; FW, floppy wings; WS, wing scales; Wi, wing. Scale bar = 1.0 mm.

The ARs were characterized by darkened pigmentation, a hard cuticle and black wings (Fig. 1E). The BNs had wing buds, a light brown cuticle and dark brown stripes on their heads (Fig. 1B). The ANs had a light brown cuticle, dark brown stripes on their heads and floppy wings that were unsclerotized and incapable of flight (Fig. 1C). These nonfunctioning wings were shed within 1 week and left two pairs of wing scales at the mesonotum and metanotum (Fig. 1D). Compared with the ARs (Fig. 1E,F), the ANs and BNs exhibited a smaller amount of sclerotized cuticles and lighter pigmentation.

The presence of wings might lead to the conclusion that the *R. labralis* ANs are alate adults, but their overall appearance is more similar to that of secondary reproductives (neotenic reproductives). Additionally, our previous study demonstrated that *R. labralis* ANs and BNs are not able to survive in the absence of workers, whereas paired alate adults are able to create new colonies without the help of workers^4^,^5^. Therefore, ANs are more similar to neotenic reproductives in terms of morphology and behaviour.

### Illumina sequence data and assembly

We constructed RNA-seq libraries using mRNA isolated from the three reproductive castes. A total of 404,152,188 clean-sequence reads were obtained via Hi-Seq^TM^ 2000 (Illumina) paired-end sequencing of *R. labralis*. Each sample generated more than 3G of transcriptome data. Based on the clean reads, a total of 61,953 unigenes ranging from 201 bp to 17,328 bp were assembled with the Trinity program (Supplementary Fig. 1). The mean length was 905 bp, and the N50 length was 1718 bp. Average quality values ≥ 20 were obtained for more than 93.8% of the cycles. The sample GC content was consistently approximately 44%. These results suggest that the sequencing output and quality were sufficient for further analysis.

### Functional annotation of the *R. labralis* transcriptome

**Figure 2:**
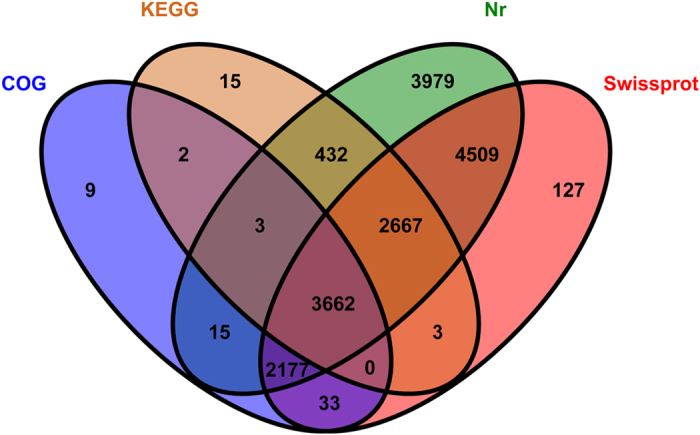
Distribution of the similarity search results illustrated with a Venn diagrams. The numbers of unique sequence-based annotations is the sum of the unique best BLASTX hits from the Nr, Swiss-Prot, COG and KEGG databases. The overlapping regions between the four circles contain the numbers of unigenes that shared BLASTX similarities with the respectively databases.

**Figure 3:**
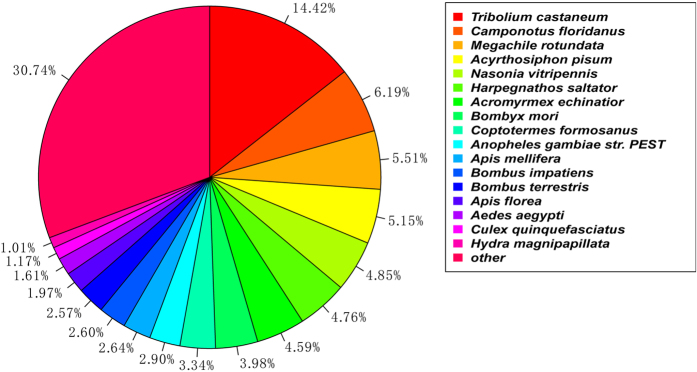
Species distribution of the BLASTX results. The species distribution of the unigene BLASTX results against the NCBI-Nr protein database with a cut-off E value  <10^−5^. The different colours represent different species.

To annotate the unigenes, we used BLASTx program (http://www.ncbi.nlm.nih.gov/BLAST/) with an E-value threshold of 1e-5 to NCBI non-redundant protein (Nr) database (http://www.ncbi.nlm.nih.gov), the Swiss-Prot protein database (http://www.expasy.ch/sprot), the Kyoto Encyclopedia of Genes and Genomes (KEGG) database (http://www.genome.jp/kegg), and the COG database (http://www.ncbi.nlm.nih.gov/COG). We annotated 61,953 unigene sequences. The Venn diagram illustrates that the number of unique sequence-based annotations is the sum of the unique best BLASTX hits from the Nr, Swiss-Prot, COG and KEGG databases. The overlapping regions among the four circles indicate the numbers of unigenes that shared BLASTX similarities in the respective databases (Fig. 2). 17,444 unigenes (28.16% of the *R. labralis* unigenes) had significant matches in the Nr database, 13,178 unigenes (21.27%) had significant matches in the Swiss-Prot database, 5,901 unigenes (9.52%) had significant matches in the COG database, and 6,784 unigenes (10.95%) had significant matches in the KEGG database (Supplementary Table 1). Due to the relatively short lengths of the distinct gene sequences and the lack of genome information for *R. labralis,* the majority of the 61,953 assembled sequences could not be matched to known genes (71.54%).These unigenes could also be *R. labralis-*specific genes or short fragments that were mainly from the untranslated (e.g., 5′ and 3′ UTRs) or non-conserved regions of protein-coding transcripts^11^. Matches to the nr database also indicated that a large number of the *R. labralis* unigenes closely matched the sequences of *Tribolium castaneum* (14.42%), *Camponotus floridanus* (6.19%), *Megachile rotundata* (5.51%), *Acyrthosiphon pisum* (5.15%) and *Coptotermes formosanus* (3.34%). Unigenes of 17 species in the Nr database had >1% match with those of *R. labralis* (Fig. 3).

### Gene Ontology (Go), Clusters of Orthologous Groups (COG) and Kyoto Encyclopedia of Genes and Genomes (KEGG) ontology classifications

**Figure 4:**
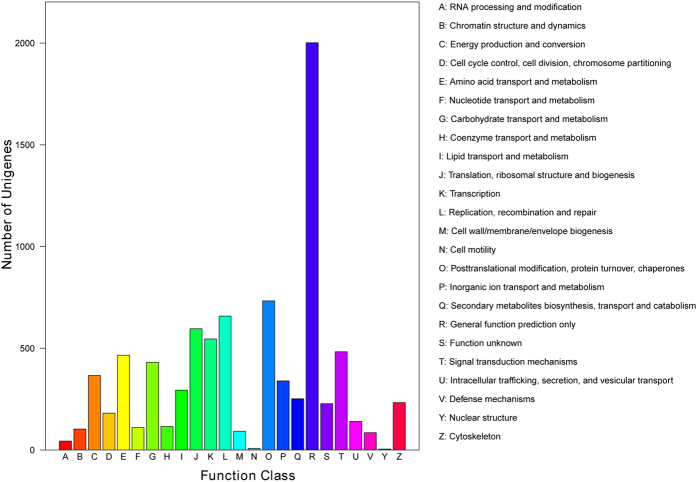
Histogram presentation of the clusters of the orthologous group (COG) classifications. A total of 5,901 unigenes were grouped into 24 COG classifications. The y-axis indicates the number of unigenes in a specific functional cluster. The legend presents the 24 functional categories.

Based on the protein annotation results from the Nr database homology search, we classified the functions of the predicted unigenes using GO, KEGG and COG analyses. In the COG functional classification, 5,901 unigenes were annotated into 24 COG categories (Fig. 4). Among these categories, general function encompassed the largest group (2,002 genes, 33.93%), followed by posttranslational modification, protein turnover, chaperones (733, 12.42%), functions in replication, recombination and repair (658, 11.15%), translation, ribosomal structure and biogenesis (596, 10.10%), transcription (546, 9.25%), amino acid transport and metabolism (466, 7.90%), carbohydrate transport and metabolism (431, 7.30%), energy production and conversion (367, 6.22%), inorganic ion transport and metabolism (340, 5.76%), lipid transport and metabolism (294, 4.98%), and secondary metabolite biosynthesis, transport and catabolism (252, 4.27%). Genes annotated as “RNA processing and modification” (44, 0.71%), “cell motility” (8, 0.14%) and “nuclear structure” (5, 0.08%) represented the smaller groups predicted by the COG.

**Figure 5:**
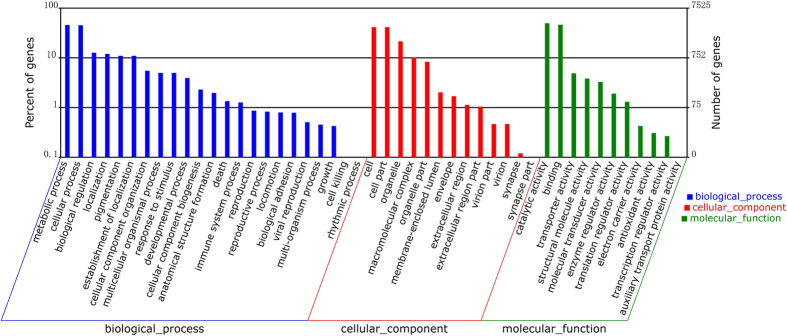
Histogram presentation of the Gene Ontology classification. The results are summarized in three main categories: biological process, cellular components, and molecular functions. The right y-axis indicates the number of genes in a category. The left y-axis indicates the percentage of a specific category of genes in the main category.

To determine the functions of the differentially expressed genes, all of the DEGs in this study were mapped to terms in the GO database. All of the annotated unigenes belonged to biological process, cellular components and molecular function clusters and were distributed into 47 categories that included catalytic activity, binding, metabolic processes, cellular processes, cell parts and organelle etc. (Fig. 5). The three main categories of GO annotations included 9,734 GO annotations (31.57%) for cellular components, 8,438 annotations (27.36%) for molecular functions and 12,665 annotations (41.07%) for biological processes.

**Figure 6:**
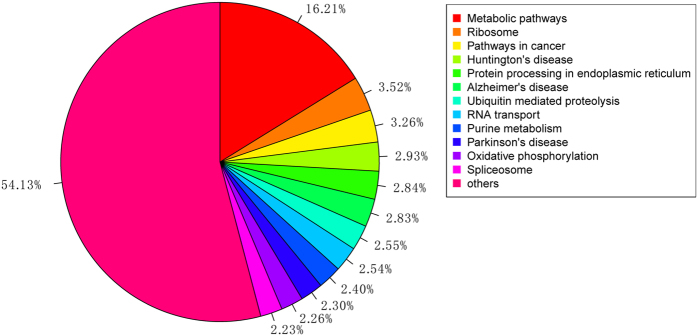
KEGG classifications of the unigenes. Six-thousand-seven-hundred-eighty-four unigenes were assigned to 236 pathways. The pathways to which more than 150 unigenes were mapped are shown.

To better understand the biological pathways that are active in *R. labralis*, we mapped the unigene sequences to the reference canonical pathways in the Kyoto Encyclopedia of Genes and Genomes (KEGG). In total, we assigned 6,784 sequences to 235 KEGG pathways (Fig. 6). The pathways that were most heavily represented by unique sequences were metabolic pathways (1,100 members), ribosomes (239 members), pathways in cancer (221 members), and protein processing in the endoplasmic reticulum (193 members). These annotations provide a valuable resource for investigating the specific processes, structures, functions, and pathways involved in reproductive differentiation.

### Differentially expressed gene (DEG) analysis in ANs, BNs and ARs

**Figure 7:**
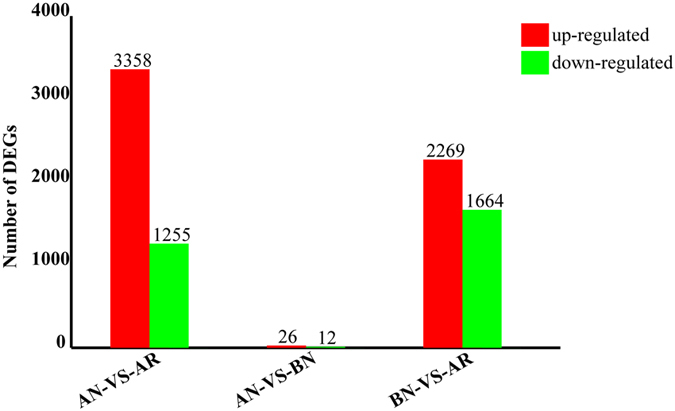
The number of the differentially expressed genes in AN, BN and AR. The x-axis indicates the three castes (AN: adultoid reproductives; BN: brachypterous neotenic reproductives; AR: alate adults). Red represent transcripts that were significant up-regulated, and green indicate that those transcripts were significantly down-regulated. The parameters FDR ≤ 0.001 and log2Ratio ≥ 1 were used as the thresholds to judge the significance of gene expression differences.

The sequence analysis and annotation for all of the unigenes in the ANs, BNs and ARs provided valuable information to analyze the *R. labralis* transcriptome. In this study, FDR ≤ 0.001 and an absolute value of log2Ratio ≥ 1 were used as filtering thresholds to identify up-regulated or down-regulated genes among ANs, BNs and ARs. As shown in Fig. 7 a total of 8,601 DEGs were screened out after a comparative analysis among ANs, BNs and ARs. Among these genes, 5,670 were identified as differentially up-regulated and 2,931 differently down-regulated. Our study showed that “AN vs BN” had the least number of DEGs compared with “AN vs AR” and “AN vs AR”, which supports our finding of ANs similar to BNs in terms of development.

To determine the biological function of DEGs among AN, BN and AR, GO classification and KEGG pathway analysis were carried out. GO classification analysis was performed on annotated transcripts using Blast2GO. The results showed that 8,601 DEGs that annotated in the GO database were categorized into 45 functional groups, including the three main GO ontologies: biological procrsses, cellular component, and molecular functions. Among these DEGs, a large number were dominant in catalytic activity, binding, metabolic process, cellular process, cell part and cell. All of the DEGs were mapped in the KEGG database to search for gene involved in the metabolic pathway, starch and sucrose metabolism, alzheimer’s disease and parkinson’s disease. KEGG pathway analysis showed that 14 pathway were significantly change (P < 0.05) in the ANs compared with BNs; 217 pathway were significantly change (P < 0.05) in the ANs compared with ARs; 38 pathway were significantly change (P < 0.05) in the BN compared with AR. KEGG analysis provides much more detailed information for understanding specific functions of a biological system during the differentiation of ANs, BNs and ARs.

### Protein-Coding Region prediction (CDS)

To further analyse the functions of the unigenes at the protein level, we predicted the protein-coding regions (CDSs) of all of the unigenes. In total, 17,612 and 2,888 unigenes were predicted using BLASTX (evalue < 0.00001) and ESTScan(version 3.0.2), respectively. The histograms in Supplementary Figs 2 and 3 illustrate the length distribution of the CDSs predicted from the BLAST and ESTScan results, respectively. In general, as the sequence length increased, the number of CDSs gradually decreased. This finding is consistent with the results of the unigene assembly.

### Cuticular protein genes related to the reproductive castes

Our analysis of the RNA-seq data identified 54 CP genes and 9 predicted CP genes that were similar to the CP genes of *Tribolium castaneum*. Of the 54 CP genes, 22 genes were classified into the CPR family, and 7 genes were classified into the CPG family (glycine-rich proteins) (Supplementary Table 2).

In the reproductive castes of *R. labralis*, of the 22 significantly expressed CPRs that could be classified, 8 were RR-1 proteins and 5 were RR-2 proteins. Our analysis revealed that the transcripts of 19 CPRs, including the 8 RR-1 and 5 RR-2 proteins, were identified in the ARs, and the transcripts of 21 CPRs, including the 7 RR-1 and 5 RR-2 proteins, were identified in both the BNs and ANs. The CPR family is by far the largest CP family in every species of arthropod and includes two major groups (RR-1 and RR-2) and a very minor RR-3 group. The vast majority of CPR genes have transcripts that are present in both the pharate and post-eclosion stages^12^,^13^,^14^. Previous studies have suggested that RR-1 proteins are associated with soft (flexible) endocuticle, whereas RR-2 proteins are more often associated with hard exocuticle.

In the present study, 7 genes that coded for CPG family members were identified in the reproductives of the termite *R. labralis*. In previous studies, CPG genes have been identified in Bombyx mori (Bombycidae) and characterized by their high glycine contents that primarily result from the repeats GGYGG and GGxGG. In Bombyx mori, CPGs contribute to larval, pupal and adult cuticles in combination with other cuticle proteins and compose the adult trachea in the wing^13^,^15^,^16^. Three glycine-rich cuticular proteins, that is, Ld-GRP1 through 3, have been identified in the Colorado potato beetle *Leptinotarsa decemlineata*, and the transcripts of Ld-GRP1 and Ld-GRP2 have been detected in the epidermal cell layer via *in situ* hybridization^17^.

### qRT-PCR verification of the CP gene expression in the ARs, ANs and BNs

**Figure 8:**
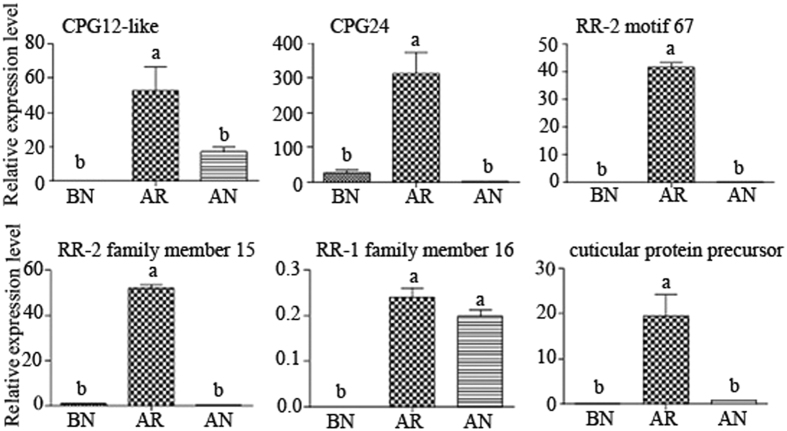
qRT-PCR analyses of the six selected genes that are involved in the cuticular layer in BN, AR and AN of *R. labralis*. The x-axis indicates the three different castes (BN: brachypterous neotenic reproductives; AR: alate adults; AN: adultoid reproductives). The letters above each bar denote significantly different groups. The significant differences were identified with the non-parametric Kruskal-Wallis test followed by Dunn’s multiple comparisons test. P-values < 0.05 were considered as significant.

We performed qRT-PCR analyses of the 6 CP genes in the ARs, ANs and BNs. The primers for the candidate unigenes, including 3 CPRs (2 RR-2s and 1 RR-1), 2 CPGs and 1 unclassified CP genes, are provided in Supplementary Table 3. Our results revealed that the CP genes displayed different expressionc patterns between the primary reproductives (ARs) and secondary reproductives (BNs and ANs): (i) the CP genes involved in cuticle sclerotization were highly expressed in the ARs and (ii) the CP genes involved in cuticle sclerotization were minimally expressed in the BNs and ANs (Fig. 8).

We demonstrated that two of the RR-2 genes were expressed at higher levels in the ARs than in the ANs and BNs. The relative expression level of RR-2 motif 67 was 165-fold and 1605-fold higher in the ARs than in the ANs and BNs, respectively (p < 0.01). The expression level of RR-2 number 15 in the ARs was 312-fold and 70-fold greater than in the ANs and BNs, respectively (p < 0.01). RR-2 containing proteins are involved in interactions with the chitin network and are predominantly found in the exocuticle^13^,^18^. N-acetyldopamine and N-β-alanyldopamine form covalent bonds with RR-2-group proteins and chitin to produce the cuticular network that is involved in sclerotization^18^. Therefore, the extremely high expression levels of the RR-2 genes in the *R. labralis* ARs are related to the hardening of the exocuticle that protects their bodies against abiotic and biotic factors after the nuptial flight.

No expression of RR-1 was detected in the BNs, and RR-1 was highly expressed in the ARs and ANs (p < 0.05). Previous studies have confirmed that RR-1 contributes to the post-ecdysial endocuticle. During the moulting process, old endocuticles are digested by chitinase and protease, and 90% of the products of this digestion are subsequently absorbed by the epidermal cells to compose new endocuticles^14^. Therefore, the expression level of RR-1 was lower than that of RR-2 and CPG. Interestingly, there was no significant difference in the expression level of RR-1 between the ARs and ANs (p > 0.05). We infer that the endocuticle is an important factor in ARs becoming primary reproductives and that the AN caste is a transitional type between the BN and AR castes in the process of evolution.

The expression levels of two of the CPG genes were significantly higher in the ARs than those in the ANs and BNs (p < 0.05). CPGs are chitin-binding proteins and compose the larval, pupal and adult cuticle in many regions of the body. In an immunocytochemical study of *Bombyx mori*, GPR2 was observed in the cuticle layer of the wing tissues and in the trachea^15^. In *Leptinotarsa decemlineata*, the CPG genes are highly induced by the insecticide azinphosmethyl (organophosphorous) 2–3 weeks after adult moulting, and the CPG gene expression level is higher in azinphosmethyl-resistant beetles than in susceptible beetles. Furthermore, the CPG genes are strongly induced by dry environmental conditions^17^. Therefore, we suggest that high expression levels of CPG genes in the ARs of termites are involved in cuticle and wing hardening and in the responses to environmental stresses.

## Materials and Methods

### Termites

Mature *R. labralis* colonies were collected from Daxingshang Temple in Xi’an City, China, in April of 2013 when the ARs were flying from the colonies, and few ANs were found in the colonies. The colonies were brought back to the laboratory, kept in plastic cases (80 × 50 × 40 cm^3^) and covered with wet soil. From August to April of the next year, the last instar nymphs (the sixth instar nymphs) appeared in the colonies (Fig. 1A).

Cuticle sclerotization often occurs in connection with moulting and begins just after a new moult^18^,^19^. To obtain ANs and BNs that had just moulted from the last instar nymphs under laboratory conditions, ten female last instar nymphs and 100 workers (NW group) were placed in a petri dish containing moist sawdust at 25 °C. One-hundred NW groups were established. The ANs and BNs were collected immediately after the last instar nymphs had moulted into ANs and BNs. In the nuptial flight season, the ARs were collected immediately after the last instar nymphs had moulted into ARs in their natal colonies. Their heads and thoraxes were immediately immersed in liquid nitrogen and stored at −80 °C for RNA extraction.

### RNA isolation, cDNA library construction and Illumina sequencing

For Illumina sequencing, the total RNA of the head-thoraxes was extracted using RNAiso Plus reagent (TaKaRa Bio. Inc., Japan) according to the manufacturer’s protocol. RNA quality was verified using an A2100 Bio-analyser (Agilent Technologies, Santa Clara, USA) and was also verified by RNase-free agarose gel electrophoresis. Next, poly (A) mRNA was isolated using oligo-dT beads (Qiagen Co., Ltd., Shanghai, China). All mRNA was broken into short fragments via the addition of fragmentation buffer. First-strand cDNA was generated using random hexamer-primed reverse transcription, followed by the synthesis of the second-strand cDNA using RNase H and DNA polymerase I. The cDNA fragments were purified using a QIA quick PCR extraction kit. These purified fragments were then washed with EB buffer for end reparation poly (A) addition and ligated to the sequencing adapters. Following agarose gel electrophoresis and the extraction of the cDNA from the gels, the cDNA fragments were purified and enriched by PCR to construct the final cDNA library. The cDNA library was sequenced on an Illumina sequencing platform (lllumina HiSeq^TM^ 2500,125 bp read length) using the paired-end technology of Gene Denovo Co. (Guangzhou, China). A Perl program was written to select the clean reads by removing the low-quality sequences (more than 50% of the bases with qualities lower than 20 in one sequence), reads with more than 5% N bases (bases unknown) and reads containing adaptor sequences.

### De novo Assembly

Transcriptome *de novo* assembly was carried out with short reads assembling program – Trinity (version 2.0.6)^20^. Trinity is a modular method and software package which combines three components: *Inchworm, Chrysalis* and *Butterfly*. Firstly, *Inchworm* assembles reads by a greedy k-mer based approach, resulting in a collection of linear contigs. Next, *Chrysalis* clusters related contigs that correspond to portions of alternatively spliced transcripts or otherwise unique portions of paralogous genes, and then builds a de Bruijn graphs for each cluster of related contigs. Finally, *Butterfly* analyzes the paths taken by reads and read pairings in the context of the corresponding de Bruijn graph, and outputs one linear sequence for each alternatively spliced isoform and transcripts derived from paralogous genes.

### Read alignments and normalization of the gene expression levels

The sequencing reads were mapped to reference sequences using SOAPaligner/soap2^21^ which is a tool that was designed for short sequence alignments. The coverage of the reads in one gene was used to calculate the expression level of that gene. Using this method, we obtained the expression levels of all of the detected genes. The reads that could be uniquely mapped to a gene were used to calculate the expression level. The gene expression level was measured by the number of uniquely mapped reads per kilobase of exon region per million mappable reads (RPKM). All expression data statistic and visualization were conduction with R package (http://www.r-project.org/).

### Differentially expressed genes (DEGs) and functional enrichment analyses

After the expression level of each gene was calculated, differential expression analyses were conducted using edgeR^22^. The false discovery rate (FDR) was used to determine the threshold for the *p* value following multiple tests, and the FDR ≤ 0.01 threshold and an absolute value of log2Ratio ≥ 1 were used to judge the significance of the gene expression differences in the analysis.

The differentially expressed genes were used for GO and KEGG enrichment analyses according to a method similar to that described by Zhang^23^. All the GO termes were classified into 47 functional groups, including three main categories: biological processes, cellular components. and molecular function. Go functional annotations of unigenes could be obtained from Nr annotation results. Go annotation of unigenes was analyzed by Blast2GO software^24^. Functional classification of unigenes was performed using WEGO software^25^. Both the GO terms and the KEGG pathways with Q-values < 0.05 are significantly enriched in DEGs.

### Quantitative Real-Time PCR

Total RNA was extracted using RNAiso Plus reagent (TaKaRa Bio. Inc., Japan) from the head-thoraxes of the adultoid reproductives, brachypterous neotenics and alate reproductives that had just moulted from the last instar nymphs (n = 5). cDNA was synthesised from 50 ng of mRNA. cDNA for qPCR was synthesized by PrimerScript RTase (Takara Bio. Inc., Japan). The quantitative reaction was performed on a LightCycler 480 software release 1.2.0.0625 (Roche Diagnostics, Switzerland) using SYBR Premix Ex Taq^TM^ II (TaKaRa Bio. Inc., Japan). All reactions in the q-PCR system were normalized using the Ct values corresponding to the actin levels according to a study of reliable reference genes for expression studies using q-PCR in *Odontotermes formosanus*^6^. The relative gene expressions were calculated using the 2^−∆∆Ct^ method^26^. All qRT-PCR experiments were repeated in three biological and three technical replications. The non-parametric Kruskal-Wallis test combined with post hoc Dunn’s multiple comparisons test was used to compare statistical differences among ARs, ANs and BNs.

## Conclusion

In *R. labralis*, the differentiation of alate adults, brachypterous neotenics and adultoid reproductives from the last instar nymphs represents a promising model system for specifying the developmental mechanism that produces the reproductive phenotypes of termites. Our study constructed transcriptome libraries of the alate adults, adultoid reproductives and brachypterous neotenic reproductives and compared the expression levels of cuticular protein genes between these three reproductive castes. This study has addressed a gap in genetic information regarding *Reticulitermes* and provided comprehensive sequence resources that are available for elucidating the molecular mechanisms that underlie reproductive caste differentiation in the termite. Additionally, our findings suggest that the alate adults might increase cuticular component deposition after moulting from the last instar nymph to adapt to new or changing environments and that the development of a reproductive individual into a primary or secondary reproductive is controlled by the expression of cuticular protein genes that are involved in exocuticle sclerotization.

## Additional Information

**How to cite this article**: Su, X. *et al*. Characterization of the transcriptomes and cuticular protein gene expression of alate adult, brachypterous neotenic and adultoid reproductives of *Reticulitermes labralis. Sci. Rep.* **6**, 34183; doi: 10.1038/srep34183 (2016).

## Supplementary Material

Supplementary Information
