# Supplementary material for: Characterization of the transcriptomes and cuticular protein gene expression of alate adult, brachypterous neotenic and adultoid reproductives of Reticulitermes labralis
Source: Sci Rep. 2016 Sep 30;6:34183. doi: 10.1038/srep34183 (PMC5044703; doi:10.1038/srep34183)
Supplement: Supplementary Information [file srep34183-s1.pdf]

Characterization of the transcriptomes and cuticular protein gene expression of alate adult, brachypterous neotenic and adultoid reproductives of *Reticulitermes labralis*

Xiaohong Su*, He Liu, Xiaojuan Yang, Jiaoling Chen, Honggui Zhang, Lianxi Xing, Xiaojing Zhang

Key Laboratory of Resource Biology and Biotechnology in Western China (Northwest University), Ministry of Education. Shaanxi Key Laboratory for Animal Conservation, Northwest University, Xi'an,China

Supplementary information

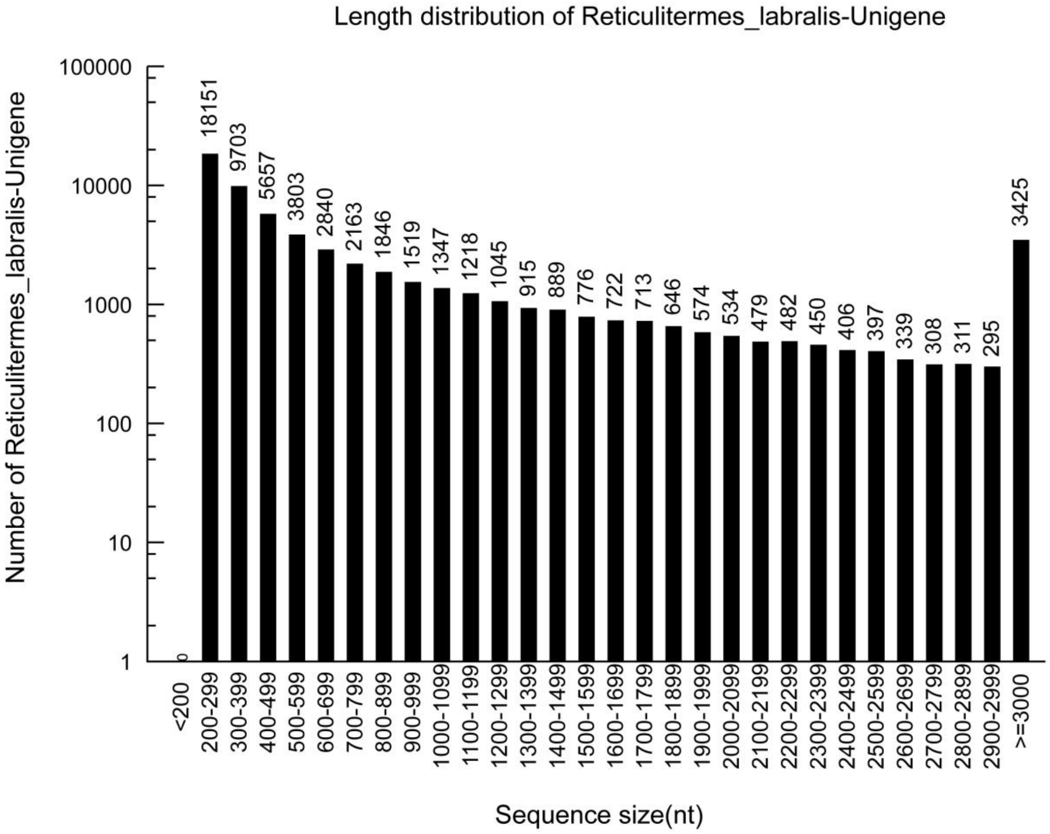

Supplementary Figure 1. Distribution of the lengths of the *R. labralis* unigenes. The histogram presents the sequence length distribution for the identified significant matches. The x-axis indicates the sequence sizes from 200 nt to >3000 nt. The y-axis indicates the number of unigenes for every given size. The results of the sequence-length matches (with a cut-off E-value of 1.0E-5) in the nr databases were greater among the longer assembled sequences.

Supplementary Table 1. Functional annotation of the *R. labralis* transcriptome. We used BLASTX to query various protein databases and to annotate 61,953 unigene sequences. All of the unigenes were annotated against the Nr, Swiss-Prot, COG, and KEGG databases.

Total Unigenes	Nr	Swissprot	KEGG	COG	Annotation genes	Without annotation gene
61,953	17,444	13,178	6,784	5,901	17,633	44,320

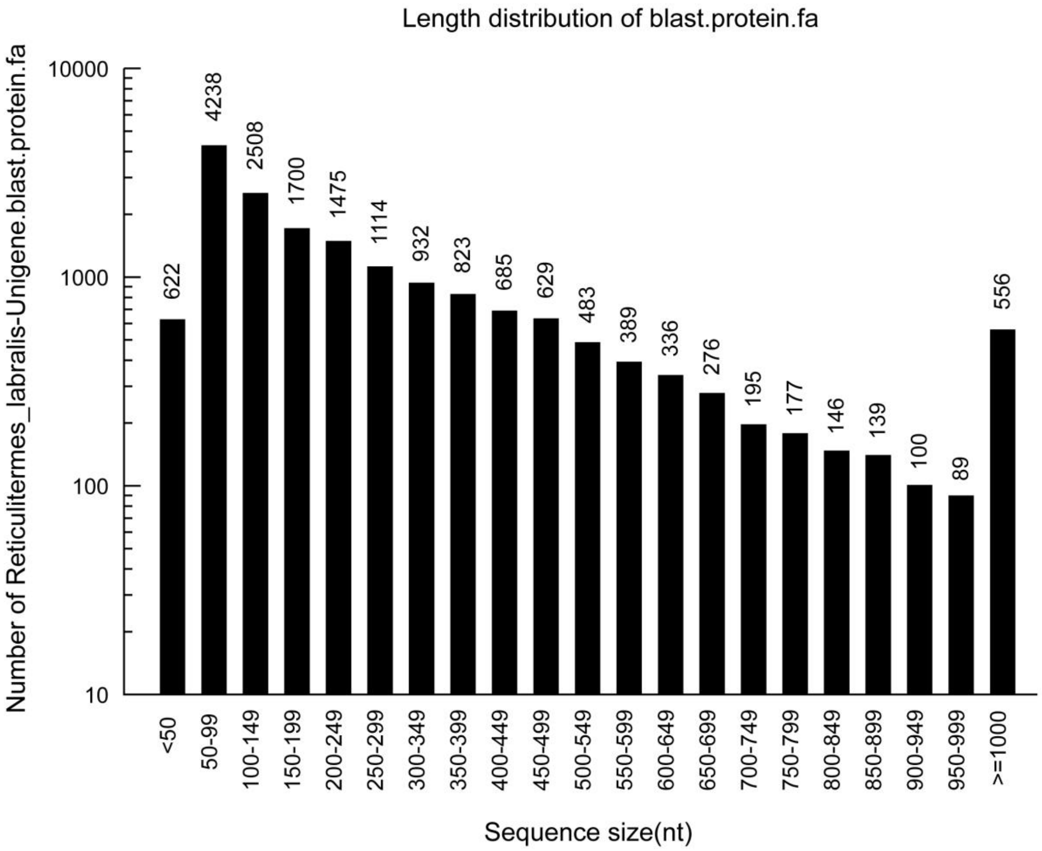

Supplementary Figure 2. Length distribution of Protein-Coding Region prediction(CDS) from BLAST. 17,612 unigenes were predicted using BLASTX. Histogram presentation of sequence-length distribution for significant matches that was found.The x-axis indicates squence size from 0 nt to >1000 nt .The y-axis indicates the number of unigenes for every given size.

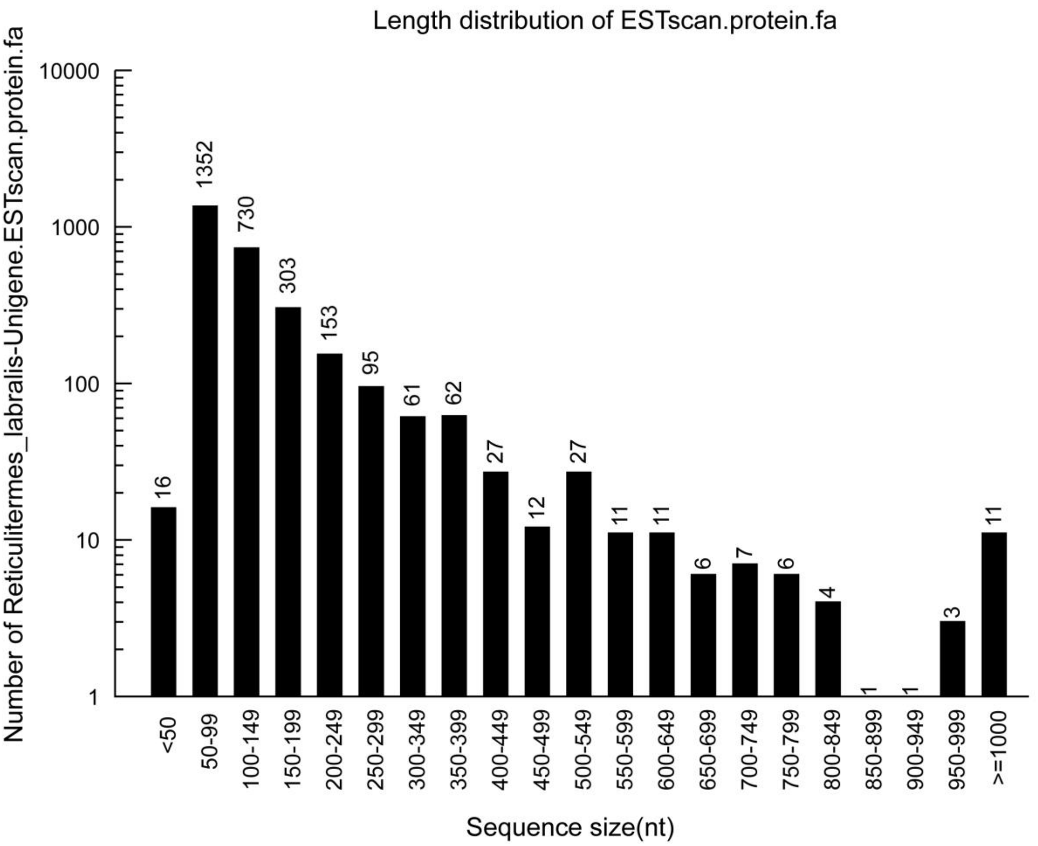

Supplementary Figure 3. Length distribution of Protein-Coding Region prediction(CDS) from ESTcan. 2,888 unigenes were predicted using ESTScan. Histogram presentation of sequence-length distribution for significant matches that was found.The x-axis indicates squence size from 0 nt to >1000 nt .The y-axis indicates the number of unigenes for every given size.

Supplementary Table 2. The analysis of the RNA-seq data identified 54 CP genes and 9 predicted CP genes in alate adults (ARs), brachypterous neotenics (BNs) and adultoid reproductives (ANs) . The plus signs (+) indicated that the CP genes were detected in ANs, BNs and ARs. The minus sign (—) indicated that the CP genes were not detected in ANs, BNs and ARs.

Unigene ID	Annotation	ANs	BNs	ARs
Unigene 0002385	cuticular protein RR-1 motif 32 precursor [*Bombyx mori*]	—	—	+
Unigene 0039759	cuticular protein RR-1 family member 16 precursor [*Nasonia vitripennis*]	+	+	+
Unigene 0018361	cuticular protein RR-1 motif 12 precursor [*Bombyx mori*]	+	+	+
	cuticular protein RR-1 motif 32 precursor			

Unigene 0029456	[*Bombyx mori*]	+	+	+
Unigene0043474	cuticular protein RR-1 motif 32 precursor [*Bombyx mori*]	+	+	+
Unigene 0028681	cuticular protein RR-1 motif 47 precursor [*Bombyx mori*]	+	+	+
Unigene 0020836	cuticular protein RR-1 motif 32 [*Antheraea yamamai*]	+	+	+
Unigene 0047831	cuticular protein RR-1 motif 32 precursor [*Bombyx mori*]	+	+	+
Unigene 0019213	cuticular protein RR-2 family member 15 precursor [*Nasonia vitripennis*]	+	+	+
Unigene 0033994	cuticular protein RR-2 motif 140 [*Danaus plexippus*]	+	+	+
Unigene 0005254	cuticular protein RR-2 motif 126 precursor [*Bombyx mori*]	+	+	+
Unigene0030153	cuticular protein RR-2 motif 67 precursor [*Bombyx mori*]	+	+	+
Unigene 0017956	cuticular protein RR-2 motif 126 [*Antheraea yamamai*]	+	+	+
Unigene 0011220	cuticular protein CPG12-like precursor [*Acyrthosiphon pisum*]	+	+	+
Unigene 0026523	cuticular protein CPG12-like precursor [*Acyrthosiphon pisum*]	+	+	+
Unigene 0025235	cuticular protein CPG12-like precursor [*Acyrthosiphon pisum*]	+	+	+
Unigene 0013033	cuticular protein CPG24 [*Papilio xuthus*]	+	+	+
Unigene 0043086	cuticular protein CPG24 [*Danaus plexippus*]	+	+	+

Unigene 0024095	cuticular protein PpolCPG4 [*Papilio polytes*]	+	+	+
Unigene 0016662	cuticular protein PpolCPG24 [*Papilio polytes*]	+	+	+
Unigene 0030156	cuticular protein CPR71 [*Papilio xuthus*]	+	+	+
Unigene 0042874	cuticular protein CPR76a [*Papilio xuthus*]	+	+	+
Unigene 0022576	cuticular protein PxutCPR32 [*Papilio xuthus*]	+	+	+
Unigene 0061252	cuticular protein PxutCPR146, partial [*Papilio xuthus*]	+	+	+
Unigene 0042871	cuticular protein PxutCPR129 [*Papilio xuthus*]	+	+	+
Unigene 0014048	cuticular protein PxutCPR129 [*Papilio xuthus*]	+	+	+
Unigene 0058898	cuticular protein PxutCPR129 [*Papilio xuthus*]	+	+	—
Unigene 0026263	cuticular protein PxutCPR129 [*Papilio xuthus*]	+	+	—
Unigene 0030155	cuticular protein PpolCPR67A [*Papilio polytes*]	+	+	—
Unigene 0020910	cuticular protein precursor [*Tribolium castaneum*]	+	+	+
Unigene 0037905	cuticular protein precursor [*Tribolium castaneum*]	+	+	+
Unigene 0028472	cuticular protein precursor [*Tribolium castaneum*]	+	+	+
Unigene 0032844	cuticular protein precursor [*Tribolium castaneum*]	+	+	+

Unigene 0028844	cuticular protein precursor [*Apis mellifera*]	+	+	+
Unigene 0046653	cuticular protein analogous to peritrophins 3-B precursor [*Acyrthosiphon pisum*]	+	+	+
Unigene 0030328	cuticular protein analogous to peritrophins 3-E [*Tribolium castaneum*]	+	+	+
Unigene 0033717	cuticular protein analogous to peritrophins 1-H precursor [*Tribolium castaneum*]	+	+	+
Unigene 0019965	cuticular protein analogous to peritrophins 3-A1 precursor [*Tribolium castaneum*]	+	+	+
Unigene 0035734	cuticular protein analogous to peritrophins 3-A2 precursor [*Tribolium castaneum*]	+	+	+
Unigene 0017559	cuticular protein analogous to peritrophins 1-D precursor [*Tribolium castaneum*]	+	+	+
Unigene 0038685	cuticular protein analogous to peritrophins 1-I precursor [*Tribolium castaneum*]	+	+	+
Unigene 0021234	cuticular protein analogous to peritrophins 1-B [*Tribolium castaneum*]	+	+	+
Unigene 0027022	cuticular protein analogous to peritrophins 1-E precursor [*Tribolium castaneum*]	+	+	+
Unigene 0046464	cuticular protein analogous to peritrophins 1-A [*Tribolium castaneum*]	+	+	+
Unigene 0010721	cuticular protein analogous to peritrophins 1-F precursor [*Tribolium castaneum*]	+	+	+
Unigene 0059039	cuticular protein [*Tenebrio molitor*]	+	+	+
Unigene 0025435	cuticular protein [*Tenebrio molitor*]	+	+	+

Unigene 0045502	cuticular protein hypothetical 28 [*Danaus plexippus*]	+	+	+
Unigene 0014401	cuticular protein hypothetical 33 precursor [*Bombyx mori*]	+	+	+
Unigene 0018309	cuticular protein hypothetical 33 precursor [*Bombyx mori*]	+	+	+
Unigene 0057233	cuticular protein-like precursor [*Acyrthosiphon pisum*]	+	+	+
Unigene 0029348	cuticular protein 6 precursor [*Apis mellifera*]	+	+	—
Unigene 0051217	cuticular protein 76Bb [*Drosophila melanogaster*]	+	+	—
Unigene 0061154	cuticular protein 50Ca [*Drosophila melanogaster*]	—	+	—
Unigene 0007627	PREDICTED: similar to cuticular protein 23, RR-1 family (AGAP005998-PA) [*Tribolium castaneum*]	+	+	+
Unigene 0006513	PREDICTED: similar to cuticular protein [*Tribolium castaneum*]	+	+	+
Unigene 0039173	PREDICTED: similar to cuticular protein 127, RR-1 family (AGAP000344-PA) [*Tribolium castaneum*]	+	+	+
Unigene 0022621	PREDICTED: similar to Cuticular protein 62Bc CG1919-PA [*Tribolium castaneum*]	—	+	+
Unigene 0022956	PREDICTED: similar to Cuticular protein 49Aa CG30045-PB [*Tribolium castaneum*]	+	+	+
Unigene 0042254	PREDICTED: similar to Cuticular protein 97Ea CG6131-PA [*Tribolium castaneum*]	+	+	+
Unigene 0025436	PREDICTED: similar to cuticular protein [*Tribolium castaneum*]	+	+	+
Unigene 0013679	PREDICTED: similar to Cuticular protein 62Bc CG1919-PA [*Tribolium castaneum*]	+	+	+

Unigene 0029347	PREDICTED: similar to Cuticular protein 62Bc CG1919- PA [*Tribolium castaneum*]	+	+	+

Supplementary Table 3. The six selected genes and their primers used in qRT-PCR analyses .

Unigeng ID	Annotation	Primera sequences
	Beta-actin (reference gene)	Forward:AGCGGGAAATCG TGCGTGAC Reverse:CAATGGTGATGAC CTGGCCAT
Unigene0011220	Cuticular protein CPG12-like precursor [Acyrthosiphon pisum]	Forward:CCTGTGAAAGTCC CGTACAA Reverse:CGACGAAATAGGG ATGAGG
Unigene0020910	Cuticular protein precursor [Tribolium castaneum]	Forward:CAGTCAGTACCAC GCACAGG Reverse:CCGTCGATGTAGG AGTAGCC
Unigene0016662	Cuticular protein PpolCPG24 [Papilio polytes]	Forward:GGTCTGGCTACGG TTATGGA Reverse:TAAGGCACGGGTT TCTCG
Unigene0039759	Cuticular protein RR-1 famliy member 16 precursor [Nasonia vitripennis]	Forward:CGGAGCAGGAAA CAC AGGT Reverse:ACAAAGCCATTCT CGTCAGC
Unigene0030153	Cuticular protein RR-2 motif 67 precursor [Bombyx mori]	Forward:AGTCGAGTACAC CGCTGACC Reverse:AGAGGAGCAGCC ACCTTAGC
Unigene0019213	Cuticular protein RR-2 famliy member 15 precursor [Nasonia vitripennis]	Forward:ACTCCCAGTATGA CCGCAAC Reverse:GCCATCGGACTCT ACAACACT
